# The paradox of action: Trajectories in distress, meaning, and gene regulation following self-directed activist engagement among Black women

**DOI:** 10.1016/j.ssmmh.2026.100654

**Published:** 2026-05-17

**Authors:** Taylor A. Geyton, Steven W. Cole

**Affiliations:** a San Diego State University, 5500 Campanille Dr., San Diego, CA, 92181, USA; b University of California Los Angeles, School of Medicine, 10833 Le Conte Ave, Los Angeles, CA, 90095, USA

## Abstract

**Background:**

Black women have a long history of engaging in activism and community advocacy as both a political imperative and a survival strategy (Hill Collins, 2000; Ransby, 2003). Activism may confer psychological benefits such as increased meaning in life, identity affirmation, and social connection (Hope et al., 2019; Klar and Kasser, 2009); simultaneously, activism can involve sustained stress exposure, burnout, and heightened vulnerability to surveillance and (re)traumatization (Gorski, 2019). Biological indicators offer one avenue for examining how social conditions are embodied particularly when psychosocial experiences involve chronic threat, vigilance, and cumulative stress exposure. Given growing attention to activism as a health-relevant practice, this study is framed though an intersectional and critical medical anthropology lens to explore how activism may relate to both psychosocial and biological stress processes among Black women.

**Methods:**

Using an embedded mixed-methods pilot design, this study evaluated the feasibility of assessing psychosocial indicators and the Conserved Transcriptional Response to Adversity (CTRA) at baseline (T1) and ~2-month follow-up (T2) during a self-selected intentional activism period among Black women, with baseline semi-structured interviews contextualizing interpretation of T1→T2 patterns. Participants (N = 29; M age = 39.4 years, SD = 11.7; range = 21–63) completed online surveys, a baseline interview, and coached dried blood spot (DBS) collection at T1, then completed follow-up surveys and DBS collection at T2. Analyses followed a three-part workflow: (1) feasibility metrics (enrollment/retention and useable DBS returns), (2) within-person quantitative change (paired-samples t tests for psychosocial measures; mixed-effects linear models of a 53-gene CTRA set), and (3) thematic analysis of baseline interviews with integration via joint display (convergence/complementarity/divergence).

**Results:**

Procedures supported remote biobehavioral assessment; useable DBS/CTRA data were available for most participants (T1 n = 28; T2 n = 27). Psychosocial scores decreased from T1 to T2 for psychological distress, activist orientation, and meaning in life (presence and search), with activist identity/commitment also decreasing but not reaching conventional thresholds. At the sample level, mean CTRA showed no net change from T1 to T2; however, within-person increases in activist identity/commitment and increases in search for meaning were associated with more favorable CTRA change. Baseline qualitative themes (Activism’s Paradox; Identity Dialectics; Resistance and Restoration; Community-Rooted Well-being) highlighted co-occurring strain, meaning, identity negotiation under misogynoir, and relational coping infrastructure.

**Conclusion:**

Findings support feasibility and provide hypothesis-generating evidence that biobehavioral responses may be contingent on identity and meaning processes rather than uniform mean shifts in a heterogeneous activism window.

## 1. Introduction

Black women in the United States experience profound health inequities, including maternal mortality rates three times higher than non-Hispanic white women even when controlling for income and education, as well as elevated rated of disordered sleep, obesity, anemia, and cardiovascular disease (CVD), (Petersen, 2019; Chinn et al., 2021). These outcomes reflect the “weathering” of the body under pervasive social and clinical inequity (Geronimus et al., 2006), yet little is known about how Black women’s intentional activism relates to the psychosocial and biological processes through which weathering unfolds.

Misogynoir, the intersection of racist and sexist oppression, necessitates chronic vigilance and coping strategies that yield a cumulative physiological burden and chronic stress among Black women (Ellis et al., 2019; Woods-Giscombe et al., 2016). The strong Black woman schema (SBWS) reflects one such strategy, involving emotional suppression and socially imposed or internalized caregiving expectations (Geyton et al., 2022). Although often framed culturally as adaptive, SBWS internalization carries significant physical and psychological health risks (Geyton et al., 2022). Activism may represent a different coping pathway by translating individual burden into collective action, yet it also exposes Black women to additional demands, risks, and surveillance that may shape stress and health.

Daily stress and perceived racism have been linked to metabolic biomarkers, and disordered sleep, illustrating how social inequality becomes embedded in regulatory systems (Jackson et al., 2020; Vines et al., 2007). Moving beyond descriptive disparities requires the application of a theoretical framework that accounts for the overlapping power structures governing these embodied processes.

### 1.1. Theoretical Foundations: a critical black feminist and structural embodiment lens

Intersectionality and critical theory together offer a critical Black feminist lens (CBF) for examining how racism and sexism together, shape exposures, constraints, and health-relevant behaviors beyond single-axis models (Bowleg, 2012; Collins, 2000; Crenshaw, 1989; Hankivsky and Christoffersen, 2008). This lens situates Black women’s coping at the intersection of misogynoir and structural constraint while centering resistance within oppressive contexts (Freire et al., 2014; Kincheloe and Mclaren, 2011).

Black feminist health science studies extend CBF to the domain of health and mental health by centering Black women’s perceptions and examining how historical and institutional forces shape activism and stress responses (Bailey and Peoples, 2017).

Complementarily, Critical Medical Anthropology (CMA) offers a structural-embodiment lens that traces how structural violence directly translates macro-level conditions into illness and stress (Farmer, 2004; Baer et al., 2013)). CMA reframes activism as a culturally meaningful practice with psychosocial and physical health implications. As applied here, CMA supports mapping cultural meanings to behavior and outcomes through semi-structured interviews, embedding activism in the sociopolitical context of misogynoir, connecting Black women’s individual narratives to population-level inequities and, using findings to inform interventions that leverage activist identity and behavior for health benefit (Andreeva and Carroll, 2013; Baer et al., 2013; Uchôa and Vidal, 1994; Witeska-Młynarczyk, 2015).

Together, the CBF and structural-embodiment framework examines Black women’s activism as a historically situated coping practice with psychological and biological consequences. It conceptualizes health as shaped by interlocking systems of power, foregrounds Black women activists’ accounts of activism, stress, and wellness, and informs the design and interpretation of this embedded mixed-methods pilot study.

### 1.2. Resistance and agency

Activism can function as a historically situated response to misogynoir among Black women. Black women’s intersecting minoritized identities may foster recognition of broader social struggles, a sense of obligation to advocate for others, and commitment to collective action (Geyton, 2021). Thus, activism operates both sociopolitically and as a meaning-making practice.

This study focuses on intentional activism rather than latent or passive resistance. Hollander and Einwohner (2004) define intentional resistance as actions consciously intended to resist power, and recognized by observers as resistance. distinguishing it from action read as subversive but not intended politically by the actor. This distinction is important because deliberate, goal-oriented engagement in social change is especially relevant for examining meaning making, identity, psychological well-being, and biological embodiment.

Intentional activism is not a uniform exposure, it varies by modality, visibility, frequency, social context, and perceived risk. Some forms of activism may involve private community organizing, mutual aid, writing, education, or relational advocacy, while others may involve public protest, confrontation with state violence, or highly visible digital engagement. For Black women contemporary activism often spans physical and digital spaces. Digital activism can support community-building, identity work, rapid mobilization and counter-narratives that bypass traditional gatekeepers (Knight Steele, 2021) but also introduces risks such as repeated exposure to traumatic media, and the persistent threat of digital harassment or surveillance (Gray and Stein, 2021; Perera, 2023). These distinct exposures suggest that activisms health implication may depend on modality and perceived visibility-related risks.

### 1.3. Psychosocial pathways of activism

The relationship between activism and well-being is understood as a dual-process involving restoration and strain. Intentional engagement may support eudaimonic well-being, identity coherence, collective efficacy, and social connection; prior research suggests that activists report higher levels of flourishing and subjective vitality than non-activists (Klar and Kasser, 2009; Szymanski et al., 2023). Activism may therefore buffer the isolating effects of discrimination by offering meaning, community and sense of agency, with meaning in life, activist identity, and activist commitment may capturing potentially protective dimensions of engagement.

Conversely, the labor of resistance can incur psychological costs, including activist burnout, chronic exhaustion, and anxiety, particularly when sustained under racial threat, perceived obligation, and expectations of caretaking, strength and responsibility to community (Conner et al., 2023; Wolk et al., 2024). Activism may therefore be experienced as both empowering and depleting.

### 1.4. Biological embodiment of restoration and strain

Competing psychosocial pathways raise questions about biological embodiment. Biological indicators can help examine how social conditions, perceived threat, vigilance, and meaning-making become embedded in physiological processes. CTRA has been associated with chronic stressors such as social threat, isolation, trauma, and adversity, but its relationship to activism, particularly among Black women, remains underexplored (Cole, 2013; Slavich and Cole, 2013). Clarifying whether activism corresponds with psychosocial and transcriptional patterns in convergent or divergent ways may improve conceptual precision about when activism operates as restoration, strain, or both.

CTRA represents a molecular shift in RNA expression within immune cells, characterized by an up-regulation of pro-inflammatory genes and a down-regulation of genes involved in Type I interferon antiviral responses (Slavich and Cole, 2013). It can reflect both rapid per-cell transcriptional remodeling after stress exposure and longer-term changes in leukocyte composition (Cole, 2010, 2013). Social genomics research suggests that prosocial behaviors, well-being, and optimism may be associated with reduced CTRA expression, whereas social isolation and chronic threat may be associated with increased CTRA expression (Fredrickson et al., 2013; Moieni et al., 2020). Activism may plausibly operate through both pathways as purpose, collective connection, and belief in social change may correspond with lower CTRA expression, whereas chronic vigilance, exposure to racialized violence, digital harassment, or sustained obligation may correspond with higher CTRA expression. This ambiguity underscores the need for a mixed-methods approach that examines psychosocial and transcriptional patterns while capturing how Black women interpret the meaning, risks, and demands of activism.

### 1.5. Current study

This pilot study addresses a critical gap by examining intentional activism and the biological embodiment of stress among Black women. By integrating psychosocial measures with CTRA, the study provides feasibility data and preliminary direction-of-association estimates regarding changes in psychosocial outcomes and CTRA profiles across a two-month period of self-selected activism.

### 1.6. Research questions

Guided by this integrated framework, this embedded mixed-methods pilot study has two aims (1) to examine within-person change in psychosocial outcomes and CTRA profiles across a two-month period of self-selected activism among Black women, using baseline qualitative interviews to contextualize these patterns; and (2) to assess the feasibility of recruiting, retaining, and collecting longitudinal biological and psychosocial data from Black women activists. To address these aims, we posed the following research and feasibility questions:

#### Research questions.

(MMRQ = mixed-methods research question; QUAN = quantitative; QUAL = qualitative; INT = integration).

- MMRQ: How do Black women’s psychosocial and biological indicators change from T1 to ~2-month follow-up during self-selected activism, and how do baseline qualitative accounts of activism and well-being and how do baseline qualitative accounts of activism and well-being contextualize these patterns within structural inequality?
- QUAN RQ1: What within-person changes are observed in psychosocial outcomes including meaning in life, psychological distress, activist orientation, activist identity from T1 to T2?
- QUAN RQ2: What within-person changes are observed in CTRA profiles from T1 to T2, reflecting biological embodiment of stress and social conditions during activism?
- QUAN RQ3 (exploratory): To what extent does within-person change in CTRA covary with within-person change in psychosocial outcomes among Black women activists?
- QUAL RQ1: How do Black women activists define activism, describe prior experiences, and perceive its relationship to psychological and physical well-being within broader social and institutional environments?
- INT RQ: How do baseline qualitative themes on activism, stress, and wellbeing contextualize T1→T2 quantitative findings across psychosocial and biological indicators?

### 1.7. Feasibility questions

Feasibility objectives assessed the practicality of longitudinal, biologically informed research with Black women activists.

- FQ1: What proportion of individuals who initiated contact were eligible, enrolled, and completed T1 procedures?
- FQ2: What proportion of enrolled participants completed T2 procedures at ~2 months?
- FQ3: What proportion of participants provided useable DBS/CTRA samples at T1 and at T2?

These questions reflect the exploratory nature of this pilot and inform future research on the health relevance of activism under conditions of structural adversity.

## 2. Methods

### 2.1. Embedded mixed methods design

We employed an embedded mixed-methods pilot design to examine potential divergence between self-reported well-being and biological indicators. At T1, participants completed standardized psychosocial measures and a qualitative interview on activism histories, meanings, and perceived or anticipated impacts of activism on well-being; at T2 (~2 months), they repeated psychosocial measures and provided a second DBS sample. Participants were adults (M age = 39.4; range = 21–63), an age range in which role demands, sociopolitical context, and cumulative stress exposure may shape activism engagement and health-relevant outcomes.

A primary challenge in activism research and social genomics is that empowerment and distress may coexist; someone may report high levels of purpose while their body remains in a state of high inflammatory alert. Baseline qualitative interviews allowed us to define activism as participants enacted it, identify anticipated costs and benefits, and contextualize T1 to T2 quantitative change.

Quantitative and qualitative data were analyzed using distinct procedures and integrated at interpretation to identify convergence, complementarity, and divergence across psychosocial and biological indicators. This integrated approach also ensured that the biological data is not stripped of its sociopolitical context.

#### 2.1.1. Operational definition of intentional activism

Drawing on Hollander and Einwohner (2004), intentional activism was defined as a self-identified act undertaken to resist, challenge, or seek change in a sociopolitical condition. Participants were instructed to engage in at least one act they understood as activism and aligned with their sociopolitical commitments, distinguishing it from resistance that occurs incidentally or without conscious intent.

### 2.2. Recruitment and eligibility

This study used non-probability purposive and criterion-based recruitment. Eligible participants were self-identified Black women, aged 18 or older, residing in the United States, and self-identifying as activists. Individuals receiving immunosuppressive treatment were excluded due to potential effects on inflammatory and transcriptional processes. Participants were recruited through online advertising, paid social media advertisements, virtual Black activist spaces, activist organization listservs, and snowball sampling.

Given the risk of online survey fraud and the inclusion of biological sample collection, prospective participants completed a brief video-based screening prior to enrollment to confirm eligibility and to support transparency about study procedures.

### 2.3. Sample

Of 47 participants who submitted baseline surveys and passed initial eligibility screening, duplicate entries and incomplete cases (>30% missing baseline survey data) were removed; four participants withdrew after baseline procedures. The final analytic sample included 29 participants, all of whom completed T1 survey and interview procedures. Survey analyses used the full sample (N = 29), whereas DBS-derived CTRA analyses used available cases at each time point (T1 n = 28; T2 n = 27).

### 2.4. Procedures

T1 marked the start of the two-month activism period. Participants completed an online survey capturing demographic and health information and standardized psychological assessments, followed by a one-on-one semi-structured interview on definitions and experiences and perceived psychological, physiological, and meaning making/spiritual impacts of activism. At the conclusion of the interview, participants were coached through baseline DBS collection using a mail-in kit for gene expression analyses.

After the baseline interview, participants described their selected activism including planned activity, timing, modality, and perceived risk/visibility, for descriptive characterization of exposure. They were instructed to engage in their chosen activism over two months. Beyond at least one act, no restrictions were placed on form or frequency preserving authentic and accessible engagement. All participants selected activism with which they were already familiar.

**Fig. 1.**
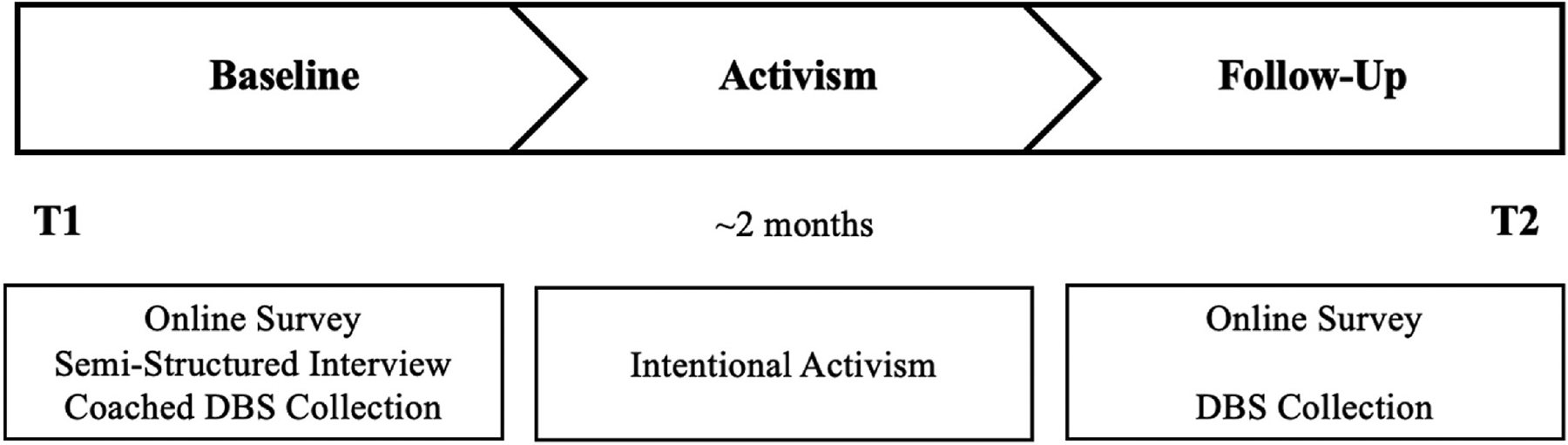
Study timeline.

At T2, initiated after email confirmation of completed activism, participants repeated standardized psychological assessments and collected and mailed a second DBS sample using the same procedures. Fig. 1 summarizes the study timline.

### 2.5. Psychosocial measures

#### 2.5.1. Health and context characteristics

At T1, participants reported date of birth, and lifetime CVD diagnosis. At both T1 and T2, participants reported neighborhood characteristics and recent DBS-relevant exposures nicotine and other substance use over the prior month, and alcohol use, THC use, and acute illness in the prior two weeks.

#### 2.5.2. psychological distress

Psychological distress was assessed using the Kessler Psychological Distress Scale (K10) (Kessler et al., 2002), a 10-item self-report scale evaluating symptoms of anxiety and depression experienced in the previous four weeks.

#### 2.5.3. eudaimonic well-being

The Meaning in Life Questionnaire (MLQ) (Steger et al., 2006) was used to measure eudaimonic well-being through two subscales: presence of meaning and search for meaning. The MLQ uses a 7-point Likert response format.

#### 2.5.4. Activist Orientation and Identity

Activist engagement was assessed using the Activist Orientation Scale (AOS) (Corning and Myers, 2002), a 32-item scale measuring attitudes and behaviors related to both conventional and high-risk activism and The Activist Identity and Commitment Scale (AICS) (Klar and Kasser, 2009), an 8-item scale adapted from Reid’s (2004) Social Identity Specific Collectivism Scale, measuring strength and salience of activist identity. Reliability for psychosocial measures is provided in Table 1.

#### 2.5.5. CTRA

Participants provided DBS samples at baseline and follow-up to assess CTRA, a biomarker profile characterized by upregulated inflammatory gene expression and downregulated antiviral gene expression in response to chronic stress (Cole, 2013). Participants received two TASSO M20 self-collection kits, return postage, and written, recorded, and verbal collection instructions. After indentifying their planned activism activity and dates, participants were coached through T1 DBS collection by video and instructed to complete and return the second DBS after two months of activism. Reminder emails at two-months included a link to the T2 survey and DBS return instructions.

After study completion, DBS samples were processed and sequenced at the UCLA Social Genomics Core Laboratory following established procedures (Snodgrass et al., 2022). Briefly, total RNA was extracted from two Tasso M20 sample matrices (~34 μL blood; Qiagen RNeasy), reverse transcribed into cDNA using a high-efficiency mRNA-targeted enzyme system (Lexogen QuantSeq 3′ FWD) and sequenced on an Illumina NextSeq instrument (Lexogen Services GmbH), all following the manufacturers’ standard protocols for low-mass RNA samples. Assays targeted 5 million sequencing reads per sample, each of which was mapped to the GRCh38 reference human transcriptome using the STAR aligner (average 98.4% mapped). Gene expression quantified as gene-mapped transcripts per million total mapped reads (TPM). TPM values were floored at 1 to suppress spurious low-range variability, log2-transformed to symmetrize distributions and stabilize variance, and analyzed by mixed effect linear models as described below.

### 2.6. Quantitative analytic approach

Paired-samples t tests compared T1 and T2 psychosocial scores \ following self-directed, intentional activism. Tests were two tailed with α = .05. Effect sizes were Cohen’s d for dependent means with 95% confidence intervals. Analyses were conducted in IBM SPSS Statistics v31.

CTRA data analyses used mixed effect linear models to quantify the change in average expression of an a priori-specified set of 53 canonical CTRA indicator genes from pre-to post-activism, and the extent to which changes in CTRA gene expression varied as a function of changes in Psychological Distress, Eudaimonic Well-Being, and Activist Orientation and Identity. These analyses contrasted expression of 19 canonical pro-inflammatory gene transcripts (*IL1A, IL1B, IL6, IL8, TNF, PTGS1, PTGS2, FOS, FOSB, FOSL1, FOSL2, JUN, JUNB, JUND, NFKB1, NFKB2, REL, RELA*, and *RELB)* with 34 genes involved in Type I IFN responses (*GBP1, IFI16, IFI27, IFI27L1–2, IFI30, IFI35, IFI44, IFI44L, IFI6, IFIH1, IFIT1–3, IFIT5, IFIT1L, IFITM1–3, IFITM4P, IFITM5, IFNB1, IRF2, IRF7–8, MX1–2, OAS1–3, OASL, IGJ, IGLL1,* and *IGLL3*) with the latter set sign-inverted to represent their inverse contribution to the CTRA profile (Cole, 2019). Models were fit using the R *nlme* package, with a random subject-specific intercept included to account for association among linear model residuals across genes and time points. Where indicated, analyses additionally controlled for age, acute illness, nicotine use, THC use, alcohol use, and neighborhood characteristics.

### 2.7. Qualitative data

#### 2.7.1. Qualitative data collection

Semi-structured interviews were conducted via Zoom at T1, after the baseline survey and before DBS collection. Interviews explored participants’ definitions and histories of activism perceived physical and psychological impacts of prior activism, and anticipated impacts during the subsequent two-month period. Interview questions were broad initially (e.g., “What is activism to you?“) and progressively narrowed (e.g., “In what ways does activism influence your physical or mental health?“).

A semi-structured format balanced consistency across participants with flexibility to pursue emergent, participant-defined meanings of activism and well-being. Standardized prompts were supplemented with targeted follow-up questions to clarify context, mechanisms, and subjective interpretations. For example, after early interviews indicated that well-being often included spiritual or religious meaning-making, subsequent interviews incorporated a consistent probe about spirituality or religion in activism-related well-being. Core questions were retained across interviews; additional probes captured recurrent participant-raised domains. Interviews were audio-recorded, transcribed verbatim, de-identified, and coded iteratively.

### 2.8. Rigor

#### 2.8.1. Researcher positionality

The interviewer/analyst identifies as a Black woman, scholar-activist, clinical social worker, and biobehavioral scientist whose work examines Black women’s activism, coping, identity formation, and well-being. Her clinical experience includes over a decade of care for Black women experiencing psychological distress and somatic manifestations of stress. These commitments informed the study’s focus and served as sensitizing perspectives during interviewing and analysis. Reflexive memos were maintained throughout data collection and analysis to examine how social location and assumptions could shape questioning, rapport, and interpretation. Positionality and reflexivity are core components of rigor in social work research, ensuring interpretations do not simply mirror the researcher’s pre-existing beliefs (Rogers and Allen, 2024).

#### 2.8.2. Trustworthiness

Trustworthiness was assessed using Lincoln and Guba’s (1985) criteria of credibility, dependability, confirmability, and transferability.

#### 2.8.3. Credibility and dependability

Credibility was supported through video screening, one-to-one interviews via unique Zoom links, transparent confidentiality procedures, and multi-stage transcript verification. Transcripts underwent three rounds of review: initial transcription by the principal investigator, followed by review by an undergraduate social work research assistant and a research associate, each involving audio verification. MAXQDA (v.24) was used to manage, organize, and analyze data, supporting consistency in coding and documentation of analytic decisions.

#### 2.8.4. Triangulation

Triangulation was supported through participant member checking (Birt et al., 2016). Nine participants who completed all study components reviewed preliminary themes and were invited to comment on accuracy and representation. Participants generally described the themes as aligned with their experiences; one noted that her views had shifted since T1 and that the summary reflected her earlier perspective more than her present-day interpretation.

#### 2.8.5. Reflexivity & audit trail

Reflexive memos were written throughout interviewing and analysis to examine how the interviewer/analyst’s assumptions, social location, and prior experiences could shape question phrasing, rapport, and interpretation (Finlay, 2002; Ortlipp, 2015). An audit trail documented analytic decisions, including dated memos, codebook iterations, coding decision rules, and theme development supporting confirmability and dependability by tracing the research path from raw data to findings (Carcary, 2021; Rodgers and Cowles, 1993).

## 3. Results

### 3.1. Sample

Participants (N = 29) ranged in age from 21 to 63 years (M = 39.4, SD = 11.7) and represented 25 municipalities across the United States. Recent health behaviors and DBS-relevant exposures at T1 and T2 are presented in Tables 2 and 3. Across both time points, acute illness was uncommon, other substance use was low, and participants primarily characterized their neighborhoods as urban or suburban.

At T2, participants again reported recent health behaviors and context characteristics relevant to the DBS window. Alcohol use frequency over the prior two weeks averaged 2.31 (SD = 2.90; range = 0–10), and THC use frequency over the prior two weeks averaged 1.93 (SD = 5.13; range = 0–20). No participants reported other substance use over the prior month (M = .00, SD = .00; range = 0–0). Three participants (10.3%) reported nicotine use during the prior month, and 2 participants (6.9%) reported an acute illness (e.g., cold, flu, COVID-19) during the prior two weeks. Neighborhood type at T2 was reported as urban (37.9%), suburban (31.0%), or rural (13.8%) and represented 25 municipalities across the United States; neighborhood data were missing for 5 participants (17.2%). Follow up sample characteristics are presented in Table 3.

Participants’ self-selected activism spanned policy advocacy, digital activism, community events, public education, workplace/institutional advocacy, organizing, capacity-building, and mutual aid/volunteering. As shown in Table 4, secondary tags were not mutually exclusive and captured multi-component activities.

### 3.2. Qualitative results

Thematic analysis yielded four themes describing activism as both taxing and restorative, and identity and community as influential to perceived wellbeing. Table 5 provides illustrative quotes for each of the subsequent themes.

#### 3.2.1. Activism’ s paradox

Participants described activism as both emotionally taxing and morally affirming. They noted the physical and mental costs of continually responding to injustice, including burnout, somatic strain, fatigue, and psychosomatic discomfort. At the same time, activism generated affirmation, hope, and empowerment, producing emotional ambivalence as participants moved between hope and despair, resilience and vulnerability.

#### 3.2.2. The dialectics of activist identity

Participants described tensions between self-perception and how they were read in activist roles, particularly as Black women navigating racialized, gendered, and professional spaces. Assertiveness was often misread as aggression, even when grounded in clarity and integrity. These dialectics reflected broader structural and identity-based negotiations, requiring participants to balance values, emotional labor, and self-protection within institutions that often misinterpreted or resisted their presence.

#### 3.2.3. Activism as restoration and resistance

Despite its burdens, activism functioned as a source of healing, meaning, and spiritual grounding. Participants described renewal through collective and expressive action, engagement with meaningful issues and communities, and the restorative value of speaking up. In this sense, activism operated as both resistance to oppression and an investment in long-term well-being.

#### 3.2.4. Community-Rooted Well-being

Participants emphasized that wellness was forged through relationships, shared struggle, and collective care. Shared experience, professional dialogue, affirmation, recognition, feedback, and interpersonal care emerged as health-promotive resources that helped participants manage the emotional weight of activism.

Together, these themes depict activism as an embodied, relational, and identity-bound practice that was experienced as both taxing and restorative. Participants described emotional and physical tolls alongside clarity, pride, moral conviction, identity negotiation, meaning-making, and community-based care. Across narratives, activism was not a discrete activity but a practice that required energy, yielded insight, and sustained connection.

To contextualize exemplar quotations while protecting participants, Table 6 provides limited descriptors for quoted participants, including pseudonym, age, region, and general activism modality/category.

### 3.3. Quantitative results

#### 3.3.1. Psychosocial

Paired-samples t tests comparing T1 scores with T2 scores over the two-month period of self-directed, intentional activism was associated with decreases in AOS, K10, and both MLQ subscales (see Table 7). The largest standardized mean change was observed for K10 (dz = .59, p = .004), followed by AOS (dz = .53, p = .008). Both MLQ-P (dz = .41, p = .034) and MLQ-S (dz = .51, p = .011) declined from T1 to T2. AICS also decreased, but this change did not reach statistical significance (p = .088, dz = .33). Two-tailed p values and 95% confidence intervals for mean differences and effect sizes are presented in Table 7.

#### 3.3.2. CTRA

Consistent with the Paradox of Activism (i.e., a mixture of positive and negative impacts), there was no net change in the CTRA gene expression profile from pre-to post-activism across the sample as a whole (simple change: −.001 log2 mRNA abundance ± standard error .071, *p* = .986; additionally controlling for age, acute illness, nicotine use, alcohol use, THC use, and neighborhood characteristics: −.061 ± .080, *p* = .443). However, those participants who experienced more positive psychological responses to activism showed more favorable changes (i. e., reductions) in CTRA gene expression in a number of respects. Specifically, those who showed higher levels of Activist Identity and Commitment over time also showed greater declines in CTRA gene expression over time (−.124 per SD of AICS ± .041, *p* = .004). These effects were driven by a modest CTRA association with the Identity subscale of the AICS (−.084 ± .042, *p* = .048) and a CTRA association with the AICS Commitment subscale (−.168 ± .046, *p* < .001). Similarly, those who showed increases in search for meaning in life from pre-to post-activism also showed reductions in CTRA gene expression (MLQ-Search: −.108 ± .044, *p* = .014). By contrast, pre-to post-activism changes in the presence of meaning in life were not associated with CTRA changes over time (MLQ-Presence: −.008 ± .046, *p* = .866).

Activist Identity and Commitment was associated with greater levels of psychological distress (K10 correlation with AICS: *r* = .37). However, those who showed greater increases in psychological distress over time from pre-to post-activism also showed greater decreases in CTRA gene expression over time (−.120 ± .042, *p* = .005), consistent with the possibility that the experienced “struggle” of activism may still provide more favorable stress-related gene regulation. Model estimates are summarized in Table 8.

### 3.4. Integrated findings

Across data sources, results supported a mixed pattern in which activism was experienced as simultaneously taxing and restorative. Qualitative themes emphasized co-occurring strain and alignment (Activism’s Paradox), identity-based negotiation (Identity Dialectics), restoration through action (Resistance and Restoration), and protection through relational ties (Community-rooted Wellbeing). Consistent with this complexity, psychosocial indicators showed T1→T2 decreases in K10, AOS, MLQ-P, and MLQ-S, with a non-significant decrease in AICS (Table 7). In contrast to mean-level psychosocial change, CTRA showed no net T1→T2 change at the sample level (Table 8, Panel A), but within-person shifts in specific psychosocial domains covaried with CTRA change (Table 8, Panel B). Given the pilot sample, integration emphasizes pattern direction and uncertainty rather than definitive convergence or divergence.

#### 3.4.1. Convergence

Convergence was most evident for activism “costs”: qualitative accounts of depletion, ambivalence, and burnout map onto the observed declines in AOS and both MLQ subscales, suggesting that engagement across a two-month window may be accompanied by reduced orientation toward activism and reduced eudaimonic indicators for some participants. At the same time, qualitative accounts of restoration and identity alignment help interpret why biological change was not uniform. Although there was no average change in CTRA, participants who showed more favorable psychosocial trajectories in targeted domains (higher AICS; higher MLQ-S) showed greater reductions in CTRA, indicating heterogeneity consistent with the qualitative depiction of activism as both strain and restoration rather than a single-direction exposure.

#### 3.4.2. Divergence

Divergence was also present: despite narratives describing strain, K10 decreased on average, and increases in K10 covaried with reductions in CTRA. This pattern suggests that K10 is not a simple proxy for biological threat in this context and may reflect “struggle” or mobilization during activism that co-occurs with more favorable transcriptional shifts for some participants. Together, the integrated findings support activism as a biobehavioral paradox: psychosocial burden and psychosocial benefit can co-occur, and transcriptional responses may align more strongly with identity/commitment and meaning-related processes than with mean-level distress or mean-level activism engagement. Table 9 is a joint display of integrated data.

## 4. Discussion

### 4.1. Feasibility

Using an embedded mixed-methods pilot design, this study supported the feasibility of remote psychosocial assessment, baseline qualitative contextualization, and mail-in DBS collection among Black women activists. Survey analyses included the full analytic sample (N = 29), and CTRA data were available for most participants (T1 n = 28; T2 n = 27), supporting the viability of integrating transcriptional profiling into activism research with appropriate attention to missingness and available-case inference.

This feasibility pattern aligns with evidence supporting remote, multi-modal biobehavioral data collection in community-based samples. Prior studies have reported high DBS return or processing rates in fully remote and behavioral trial protocols Reed et al. (Firkey et al., 2023; Reed et al., 2024), as well as strong adherence to intense remote biobehavioral protocols among middle aged African Americans (Nam et al., 2020). The present study’s CTRA yield is therefore consistent with evidence that complex remote assessment procedures can be feasible with adequate support structures and with available-case inference when biologically derived outcomes exhibit minor, non-systematic missingness (Cole et al., 2019).

### 4.2. Psychosocial findings

Psychosocial change suggested a mixed pattern consistent with activism’s dual status as strain and restoration. Psychological distress decreased from T1 to T2, while activist orientation, meaning in life, and activist identity/commitment declined, although the AICS decline was not statistical significant. This pattern is compatible with “relief alongside depletion”: participants may experience reduced distress while simultaneously feeling less oriented toward activism or less resourced for sustained meaning making. Prior work links activism to flourishing, vitality, and protection against mental health decline after sociopolitical threat (Klar and Kasser, 2009; Wnuk et al., 2023). while research on activist burnout and activist purpose demonstrates that moral commitment and eudaimonic potential can coexist with exhaustion, depressive symptoms, and anxiety (Gorski, 2019; Wolk et al., 2024) The present findings extend this work by suggesting that distress relief and resource depletion may co-occur within the same individuals across a brief longitudinal window.

Qualitatively, this tension was captured directly in Activism’s Paradox, where participants described activism as both draining and affirming echoing research on racial battel fatigue among women of color in activist spaces (Danquah et al., 2021). Identity Dialectics further reflected how misogynoir required ongoing identity negotiation consistent with SBWS scholarship on strength caretaking and self-silencing (Woods-Giscombé, 2010). Together, the quantitative and qualitative patterns suggest that activism among Black women under misogynoir is psychosocially complex: its costs and benefits may not move in tandem, a nuance that single-outcome or cross-sectional designs would obscure.

### 4.3. CTRA findings

At the transcriptional level, mean CTRA did not change from T1 to T2 in unadjusted or covariate-adjusted models. Given heterogeneous activism forms, intensities, and contexts, a null mean shift suggests that transcriptional responses may vary by appraisal, meaning, and identity processes rather than activism exposure alone. This aligns with Cole’s (2019) social signal transduction model, in which CTRA activity tracks CNS threat-appraisal processes more closely than discrete stressor exposure or self-reported adversity (Mehl et al., 2017). Qualitative theme similarly framed activism as contingent: participants emphasized that engagement conditions, visibility, and relational resources shaped whether activism felt restorative, burdensome, or both.

### 4.4. CTRA and psychosocial integration

Although mean CTRA did not change at the sample level, within-person change in CTRA covaried with selected psychosocial indicators. Increases in activist identity/commitment and search for meaning were associated with more favorable CTRA change while change in presence of meaning was not. This pattern aligns with social genomics research linking eudaimonic well-being to lower CTRA expressionand with work suggesting that search for meaning may be more relevant to favorable gene expression than presence of meaning (Cole et al., 2015; Fredrickson et al., 2013; Lee et al., 2024). Increases in psychological distress over time were also associated with more favorable CTRA change consistent with prior evidence that CTRA may track automatic threat-appraisal systems more closely than conscious distress (Fredrickson et al., 2013; Mehl et al., 2017). Together, these findings suggest that psychosocial and transcriptional changes may not move in lockstep; some participants may experience activism as emotionally taxing while also experiencing it as identity-consolidating, morally coherent, or socially anchored.

### 4.5. Mixed-methods integration

The integrated joint display helps clarify where the data converge, diverge, or function complementarily. For Activism’s Paradox, decreased distress co-occurred with declines in orientation/meaning while mean CTRA showed no uniform shift, suggesting that activism’s psychosocial profile may be mixed even when average transcriptional change is not detectable in a small pilot. For Identity Dialectics, psychosocial means trended downward while CTRA covariation suggested more favorable transcriptional change when activist identity/commitment strengthened, highlighting divergence between mean-level change and within-person coupling. For Resistance and Restoration, qualitative narratives of healing and meaning-making persisted alongside mean declines in meaning measures, yet favorable CTRA change was observed when search for meaning increased, supporting the idea that it may be the direction of individual meaning-making trajectories, rather than mean group change, that carries biological relevance in this window (Lee et al., 2024). Finally, Community-Rooted Well-being functioned as a complementary theme: qualitative data identified relational and collective resources as central to coping infrastructure, but the quantitative battery did not directly operationalize community support or collective coping. The pattern linking CTRA change with distress change, together with the centrality of community in qualitative narratives, suggests a priority for future measurement rather than a conclusion about mechanism.

### 4.6. Limitations

Several limitations constrain inference and clarify how these results should be read. First, this was a small, non-random pilot with limited power and a heterogeneous activism exposure window, which increases uncertainty around effect estimates and reduces interpretability of null findings at the sample level. The non-random design also limits generalizability beyond the study sample. Future research should use larger, more systematically recruited samples that are adequately powered to estimate within-person change and examine heterogeneity across activist identities, strategies, and contexts.

Second, only two time points were collected, limiting the ability to model trajectories, timing effects, or delayed responses in psychosocial or transcriptional outcomes. The observed T1 to T2 patterns therefore represent change across an approximately two-month period, but they cannot clarify whether changes emerged immediately after activism, accumulated gradually, fluctuated over time, or appeared after a delay. Future studies should incorporate multiple repeated assessments across shorter and longer intervals to better capture temporal dynamics and distinguish acute, cumulative, and delayed responses.

Third, the qualitative interview occurred at baseline only; therefore, interpretations of change rely on baseline accounts contextualizing subsequent T1→T2 patterns rather than participants’ post-period reflections. Future mixed-methods research should include follow-up interviews or brief post-activity reflections to examine how participants make sense of changes in distress, meaning, activist commitment, and biological stress-related processes over time.

Fourth, activism “dose,” context, and visibility were intentionally unconstrained; this improves ecological validity, by allowing participants to engage in activism as it naturally occurred in their lives, but it limits attribution of patterns to specific dimensions of activism. Changes observed over time cannot be linked to a specific type, intensity, duration, risk level, or social context of activism. Future research should retain ecological relevance while measuring key dimensions of activism more systematically, including frequency, duration, perceived risk, public visibility, emotional intensity, collective versus solitary engagement, and whether the activity was experienced as restorative, obligatory, or depleting.

Fifth, although models included covariates relevant to DBS collection windows and immune activation risk (including acute illness and recent substance use), residual confounding and timing mismatch remain plausible in a short window. Gene expression and psychosocial states may be influenced by exposures that were not measured or captured with sufficient temporal precision. Future studies should use more detailed time-linked assessments of illness, medication use, sleep, menstrual cycle, substance use, major stressors, and recent activism exposure to better align covariates with biological specimen collection.

Finally, CTRA captures state-like gene expression regulation rather than a fixed genetic sequence, and because it is estimated from a canonical gene set in bulk DBS, it may be influenced by cell-type composition and unmeasured time-varying exposures. These findings should therefore be interpreted as exploratory indicators of stress-related transcriptional patterning, not as evidence of stable biological embedding or genetic change. Future studies should consider larger bio-specimen samples, repeated biological assessments, stronger control of collection timing and immune-relevant covariates, and analytic approaches that can better account for cell-type composition.

Findings involving MLQ Presence should also be interpreted with caution given its questionable baseline internal consistency (α = .66). This may reflect the small sample size, restricted variability, or the possibility that the construct of meaning operated in more relational, spiritual, or sociopolitical ways among participants than is fully captured by the MLQ Presence subscale. Future studies should further examine the psychometric performance of meaning-related measures in samples of Black women activists and consider culturally grounded measures of purpose, collective meaning, and sociopolitical identity.

### 4.7. Implications

Given the pilot design, these findings are best understood as generating directions for future research and practice considerations rather than definitive clinical or intervention recommendations. For health and mental health practitioners, the findings suggest the value of assessing activism as part of the broader social and political context of health among Black women. Rather than treating activism as uniformly protective or harmful, providers might consider how activist engagement functions for the individual, including whether it is experienced as meaningful, relational, identity-affirming, obligatory, depleting, or exposing. This may be relevant in mental health, primary care, and community health settings where stress, trauma exposure, and cardiovascular risk are already being addressed.

Future research should build on this pilot using larger, adequately powered samples and multi-wave longitudinal designs, such as three to five or more assessments, to better capture the temporal dynamics of psychosocial and transcriptional change during activism engagement. More frequent psychosocial sampling and at least one post-activism qualitative interview would help clarify how appraisal, exhaustion, meaning-making, and relational support fluctuate within activism cycles and would reduce ambiguity about directionality.

Designs that retain participant-centered relevance while improving interpretability could incorporate more standardized activism exposure parameters, including defined activity windows, minimal dose expectations, structured documentation of modality, visibility, perceived risk, and intensity, and brief post-activity check-ins. These features would allow future studies to preserve ecological validity while better identifying who benefits, who is taxed, and under what conditions biobehavioral patterns shift.

Future studies should also add direct measures of collective coping, community support, activist stressors, digital exposure, harassment, visibility, Superwoman Schema/internalized strength norms, trauma exposure, and meaning-making/spirituality, while considering activism measures developed and validated with Black participants, such as Hope et al.’s (2019) Black Community Activism Orientation scale. This work should also examine how intersecting racialized, gendered, and sexual identities shape the psychosocial costs and benefits of activism. These measures would allow tests of mechanisms highlighted in qualitative narratives but not fully captured in the current battery. Finally, larger preregistered studies with repeated CTRA sampling could more clearly separate mean change from covariation processes and evaluate whether identity, commitment, meaning-making, and collective support are robust correlates of transcriptional change across contexts.

## 5. Conclusion

In sum, this pilot supports the feasibility of integrating psychosocial assessment, baseline qualitative contextualization, and CTRA profiling in a self-selected activism paradigm among Black women. The findings reinforce activism as an embodied, identity-linked practice that can be experienced as simultaneously restorative and taxing, with biobehavioral alignment appearing more contingent on individual meaning and identity processes than on uniform mean shifts over time. These results are best interpreted as hypothesis-generating estimates that inform the next phase of design, measurement, and sampling in research on activism as a health-relevant practice under structural adversity.

## Figures and Tables

**Table 1:** Reliability of psychosocial measures.

Construct	Measure	α
Activism	Activism Orientation Scale	αT1 = .88
		αT2 = .81
	Activist Identity and Commitment Scale	αT1 = .93
		αT2 = .91
	Semi-Structured Interview	-
Well-being (psychological)	Kessler-10 item Psychological Distress Scale	αT1 = .81
		αT2 = .87
	Meaning in Life Questionnaire	αT1 = .66
	Presence of Meaning*	αT2 = .70
	Meaning in Life Questionnaire	αT1 = .91
	Search for Meaning	αT2 = .94
	Semi-Structured Interview	-
Well-being (physical)	CTRA	-
	Semi-Structured Interview	-

Note. α values are Cronbach’s alpha from the current sample at T1 and T2. Alpha is not applicable to qualitative interviews or transcriptional profiles (CTRA).

* Internal consistency for the MLQ Presence of Meaning subscale was modest at T1 (α = .66) and improved at T2 (α = .70); findings involving this subscale were interpreted cautiously.

**Table 2:** Baseline sample characteristics (n = 29).

Characteristic	M (SD)	Range
Age (years)	39.4 (11.7)	21–63
Alcohol Use Frequency (two weeks)	2.76 (3.65)	0–12
THC Use Frequency (two weeks)	3.00 (5.68)	0–20
Other Substances (one month)	.10 (.41)	0–2
Characteristic	N	%
Nicotine (one month)	8	27.6%
Acute Illness (two weeks)	2	6.9%
Neighborhood Type	N	%
Urban	13	44.8%
Suburban	12	41.4%
Rural	4	13.8%

**Table 3:** Follow-up sample characteristics.

Characteristic	M (SD)	Range
Alcohol Use Frequency (two weeks)	2.31 (2.90)	0–10
THC Use Frequency (two weeks)	1.93 (5.13)	0–20
Other Substances (one month)	.00 (.00)	00
Characteristic	N	%
Nicotine Use(one month)	3	10.3%
Acute Illness (two weeks)	2	6.9%
Neighborhood Type	N	%
Urban	11	37.9%
Suburban	9	31.0%
Rural	4	13.8%
Missing	5	17.2%

**Table 4:** Category counts for self-selected activism activities.

Primary category	Code	n	%
Policy/government advocacy	P	10	34.5
Digital activism/social media	D	5	17.2
Community event/meeting attendance	V	5	17.2
Education/facilitation/public speaking	E	4	13.8
Institutional/workplace-based action	I	2	6.9
Collective action/organizing	C	2	6.9
Capacity-building/content or course development	B	1	3.4
Mutual aid/volunteering (as primary)	M	0	.0
Secondary category	Code	n	%
Community event/meeting attendance	V	7	24.1
Education/facilitation/public speaking	E	2	6.9
Digital activism/social media	D	2	6.9
Mutual aid/volunteering	M	2	6.9
Collective action/organizing	C	1	3.4
Policy/government advocacy	P	0	.0
Institutional/workplace-based action	I	0	.0
Capacity-building/content or course development	B	0	.0

Note: Secondary tags are not mutually exclusive; a single activity could receive more than one secondary tag when clearly multi-modal (e.g., attending + testifying; identity/commitment-building + digital dissemination). Percentages reflect the proportion of participants whose activity included that secondary component.

**Table 5:** Qualitative themes and exemplar quotes on activism-related well-being among Black women.

Theme	Definition	Exemplar Quotes
Activism’s Paradox	Activism was both taxing and affirming, producing strain alongside purpose and empowerment.	*“I think dread is the main effect … healing from it was stressful … being more selective about the time that I do choose different versions and types of activism is now both empowering and stressful.“–Freedom* *“Even if it has an immediate physical … negative consequence … it promotes my well-being because I don’t have to doubt myself.“–Betty*
Identity Dialectics	Activist identity required ongoing negotiation of self-concept and others’ perceptions under misogynoir.	*“I was born Black and female, I’m gonna die Black and female … you have to get really good at navigating spaces that ain’t meant for you.“–Danielle* *“So there’s a way we have to learn how to do things like within their system, how they like to do things. So you’re not disruptive and you’re not considering you know, too verbal or whatever.” –Georgia*
Restoration & Resistance	Activism functioned as resistance and a restorative practice that supported healing and meaning.	*“And I feel better about myself, when I help others, I take pride in doing that.“–Unique* *“Activism you know, feels like it gives me purpose. It definitely makes a positive impact on my well-being. I feel you know, better just from doing it.” –Paris*
Community-rooted well-being	Well-being was sustained through relational and collective resources.	*“Talking to other people within my field … it’s comforting to see how they got through some of their issues.“-Amonie* *“… just like the shared energy of being around other people in a virtual realm, in real life in person. That also just really like makes me feel good and also just like, makes me just like slow down and pause and be like, okay, like there are other people out here” –London,*

**Table 6:** Contextual characteristics of quoted participants.

Pseudonym	Age	U.S. region	Primary activism category	Modality
Alexandria	34	West	Digital amplification	Digital
Betty	39	South	Community engagement/participatory advocacy	In-person
Amonie	23	South	Policy/institutional advocacy	Private/offline
Danielle	34	South	Creative/cultural activism	In-person
Paris	32	South	Community support/collective care	In-person
Georgia	60	West	Institutional/workplace organizing	In-person
Unique	45	West	Digital amplification	Digital
London	32	West	Mutual aid/solidarity work	In-person

**Table 7:** Changes in psychosocial measures from T1-T2.

Measure	T1 M (SD)	T2 M (SD)	ΔM	95% CI for ΔM	t	p	dz
AOS	93.41 (11.90)	73.97 (35.97)	18.44	[5.19, 31.71]	2.85	.008	.53
K10	22.03 (6.49)	16.41 (9.96)	5.62	[1.99, 9.25]	3.17	.004	.59
AICS	30.21 (6.64)	26.10 (12.98)	4.10	[−.66, 8.86]	1.77	.088	.33
MLQ-P	28.14 (4.76)	23.66 (11.96)	4.48	[.35, 8.61]	2.22	.034	.41
MLQ-S	25.00 (7.93)	20.00 (12.12)	5.00	[1.26, 8.74]	2.74	.011	.51

Note. ΔM = T1 − T2 (positive values indicate decreases from T1 to T2). dz is Cohen’s d for paired samples (standardized by the SD of the difference scores). Two-tailed p values reported.

**Table 8:** CTRA mixed-effects model results.

Panel A. Mean Change in CTRA				
Model	Term	b	SE	p
Unadjusted	Time (T2 vs. T1)	−.001	.071	.986
Adjusted	Time (T2 vs. T1)	−.061	.080	.443
Panel B. Covariation of change in CTRA with psychosocial change				
Model	Predictor (Δ, per SD)	b	SE	p
Unadjusted	AICS	−.124	.041	.004
Unadjusted	AICS Identity	−.084	.042	.048
Unadjusted	AICS Commitment	−.168	.046	<.001
Unadjusted	MLQ-Search	−.108	.044	.014
Unadjusted	MLQ-Presence	−.008	.046	.866
Unadjusted	K10	−.120	.042	.005

Note. b coefficients are in log2 mRNA abundance units. Negative b values indicate reductions (more favorable change) in the CTRA profile over time. “Adjusted” model additionally controlled for age, acute illness, nicotine use, alcohol use, THC use, and neighborhood characteristics. Predictors are scaled per SD change T1-T2. P values are reported for completeness.

**Table 9:** Joint display of integrated data.

Theme	Qual signal (T1)	Psych Δ	CTRA Δ	Int	Integrated Interpretation
Activism’s Paradox	Activism described as draining and affirming; cost coexists with alignment/empowerment.	K10 ↓; AOS ↓; MLQ-P ↓; MLQ-S ↓; AICS ↓(ns)	Mean CTRA: no net change (unadjusted; adjusted)	Cpl	Self-reported relief in distress co-occurs with declines in orientation/meaning, while CTRA shows no uniform shift and appears contingent on how activism is appraised and internalized.
Identity Dialectics	Activist identity experienced as negotiation of self-concept and others’ perceptions under misogynoir.	AICS ↓(ns)	ΔCTRA ↓ when ΔAICS ↑	Dvg	When activist identity/commitment strengthens, biological profiles shift in a more favorable direction, aligning with narratives of identity consolidation under pressure, despite mean AICS decline.
Resistance and Restoration	Activism framed as healing/meaning-making practice despite burden; renewal through engagement and emotional grounding.	MLQ-P ↓; MLQ-S ↓	ΔCTRA ↓ when ΔMLQ-S ↑; no association with ΔMLQ-P	Dvg	Restoration narratives persist even as mean orientation/meaning decline, yet CTRA decreases are observed when activist identity/commitment or search for meaning increases.
				Cnv	Restoration narratives align with CTRA decreases tied to increases in activist identity/commitment and search for meaning, suggesting embodied benefit when activism strengthens meaning/commitment.
Community-Rooted Well-being	Well-being sustained through relational and collective resources; community as coping infrastructure.	K10 ↓	ΔCTRA ↓ when ΔK10 ↑	Cpl	Community support is framed as coping infrastructure; K10 decreased overall, yet increases in distress were associated with more favorable CTRA change, suggesting a biobehavioral pattern that may depend on relational resources not directly measured.

Note. Values reflect within-person change from T1 to T2 (~2 months). CTRA models used available cases (T1 n = 28; T2 n = 27); survey-based analyses used N = 29. Integration codes: Cnv = convergence; Cpl = complementarity; Dvg = divergence; Exp = exploratory/hypothesis-generating interpretation (pilot N; heterogeneous activism exposure). ns = not statistically significant. Interpretation is hypothesis-generating due to limited power and available-case CTRA data (N = 29; CTRA n = 28 at T1, n = 27 at T2); integration codes reflect pattern direction and uncertainty rather than confirmatory inference.

## References

[R1] Andreeva T, Carroll JJ, 2013. Key theories from critical medical anthropology for public health research. part I: starting with Foucault: cultures of medicine and meanings of illness. Tobac. Control Publ. Health E. Eur 3 (1), 39–46. 10.6084/m9.figshare.729258.

[R2] Baer HA, Singer M, Susser I, 2013. Medical Anthropology and the World System: Critical Perspectives, 3Rd Ed. Bloomsbury Publishing. 10.5040/9798400684296.

[R3] Bailey M, Peoples W, 2017. Towards a Black feminist health science studies. Catalyst: Feminism, Theory, Technoscience 3 (2).

[R4] Birt L, Scott S, Cavers D, Campbell C, Walter F, 2016. Member checking: a tool to enhance trustworthiness or merely a nod to validation? Qual. Health Res 26 (13), 1802–1811. 10.1177/1049732316654870. 27340178

[R5] Bowleg L, 2012. The problem with the phrase women and minorities: Intersectionality-An important theoretical framework for public health. Am. J. Publ. Health 102 (7), 1267–1273. 10.2105/AJPH.2012.300750. PMC3477987 22594719

[R6] Carcary M, 2021. The research audit trail: methodological guidance for application in practice. Electron. J. Bus. Res. Methods 18 (2). 10.34190/JBRM.18.2.008.

[R7] Chinn JJ, Martin IK, Redmond N, 2021. Health equity among Black women in the United States. J. Womens Health 30 (2), 212–219. 10.1089/jwh.2020.8868. PMC8020496 33237831

[R8] Cole SW, 2010. Elevating the perspective on human stress genomics. Psychoneuroendocrinology 35 (7), 955–962. 10.1016/j.psyneuen.2010.06.008. 20630660 PMC2917592

[R9] Cole SW, 2013. Social regulation of human gene expression: mechanisms and implications for public health. Am. J. Publ. Health 103, S84–S92. 10.2105/AJPH.2012.301183. PMC3786751 23927506

[R10] Cole SW, 2019. The conserved transcriptional response to adversity. Curr. Opin. Behav. Sci 28, 31–37. 10.1016/j.cobeha.2019.01.008. 31592179 PMC6779418

[R11] Cole SW, Levine ME, Arevalo JMG, Ma J, Weir DR, Crimmins EM, 2015. Loneliness, eudaimonia, and the human conserved transcriptional response to adversity. Psychoneuroendocrinology 62, 11–17. 10.1016/j.psyneuen.2015.07.001. 26246388 PMC4637182

[R12] Conner JO, Crawford E, Galioto M, 2023. The mental health effects of student activism: persisting despite psychological costs. J. Adolesc. Res 38 (1), 80–109. 10.1177/07435584211006789.

[R13] Corning AF, Myers DJ, 2002. Individual orientation toward engagement in social action. Polit. Psychol 23 (4), 703–729. 10.1111/0162-895X.00304.

[R14] Crenshaw K, 1989. Demarginalizing the Intersection of Race and Sex: a Black Feminist Critique of Antidiscrimination Doctrine, Feminist Theory and Antiracist Politics, vol. 1989. University of Chicago Legal Forum, 8.

[R15] Danquah R, Lopez C, Wade L, Castillo LG, 2021. Racial justice activist burnout of women of color in the United States: practical tools for counselor intervention. Int. J. Adv. Counsell 43 (4), 519–533. 10.1007/s10447-021-09449-7. PMC8390016 34465931

[R16] Ellis EM, Prather AA, Grenen EG, Ferrer RA, 2019. Direct and indirect associations of cognitive reappraisal and suppression with disease biomarkers. Psychol. Health 34 (3), 336–354. 10.1080/08870446.2018.1529313. 30614281 PMC6492247

[R17] Farmer P, 2004. An anthropology of structural violence. Curr. Anthropol 45 (3), 305–325. 10.1086/382250.

[R18] Finlay L, 2002. Negotiating the swamp: the opportunity and challenge of reflexivity in research practice. Qual. Res 2 (2), 209–230. 10.1177/146879410200200205.

[R19] Firkey MK, Tully LK, Bucci VM, Walsh ME, Maisto SA, Hahn JA, Bendinskas KG, Gump BB, Woolf-King SE, 2023. Feasibility of remote self-collection of dried blood spots, hair, and nails among people with HIV with hazardous alcohol use. Alcohol Clin. Exp. Res 47 (5), 986–995. 10.1111/acer.15063. PMC10360030 36949025

[R20] Fredrickson BL, Grewen KM, Coffey KA, Algoe SB, Firestine AM, Arevalo JMG, Ma J, Cole SW, 2013. A functional genomic perspective on human well-being. Proc. Natl. Acad. Sci. U. S. A 110 (33), 13684–13689. 10.1073/pnas.1305419110. 23898182 PMC3746929

[R21] Freire P, Bergman Ramos M, Ramos MB, 2014. Pedagogy of the Oppressed: 30Th Anniversary Edition. Bloomsbury Academic & Professional. http://ebookcentral.proquest.com/lib/psu/detail.action?docID=1745456.

[R22] Geronimus AT, Hicken M, Keene D, Bound J, 2006. “Weathering” and age patterns of allostatic load scores among blacks and whites in the United States. Am. J. Publ. Health 96 (5), 826–833. 10.2105/AJPH.2004.060749 PMC1470581 16380565

[R23] Geyton TA, 2021. Shattered Resilience: the Identity Formation of Black Women Activists (Order No. 28410088). Available from Proquest Dissertations & Theses Global: the Humanities and Social Sciences Collection; Proquest One Academic, 2541330624. http://libproxy.sdsu.edu/login?url=https://www.proquest.com/dissertations-theses/shattered-resilience-identity-formation-black/docview/2541330624/se-2.

[R24] Gorski PC, 2019. Fighting racism, battling burnout: causes of activist burnout in US racial justice activists. Ethn. Racial Stud 42 (5), 667–687. 10.1080/01419870.2018.1439981.

[R25] Gray KL, Stein K, 2021. “We ‘said her name’ and got zucked”: black women calling-out the carceral logics of digital platforms. Gend. Soc 35 (4), 538–545. 10.1177/08912432211029393.

[R26] Hankivsky O, Christoffersen A, 2008. Intersectionality and the determinants of health: a Canadian perspective. Crit. Public Health 18 (3), 271–283. 10.1080/09581590802294296.

[R27] Hollander JA, Einwohner RL, 2004. Conceptualizing resistance. Sociol. Forum 19 (4), 533–554. 10.1007/s11206-004-0694-5.

[R28] Jackson CL, Powell-Wiley TM, Gaston SA, Andrews MR, Tamura K, Ramos A, 2020. Racial/ethnic disparities in sleep health and potential interventions among women in the United States. J. Womens Health 29 (3), 435–442. 10.1089/jwh.2020.8329. PMC7097680 32096683

[R29] Kessler RC, Andrews G, Colpe LJ, Hiripi E, Mroczek DK, Normand SLT, Walters EE, Zaslavsky AM, 2002. Short screening scales to monitor population prevalences and trends in non-specific psychological distress. Psychol. Med 32 (6), 959–976. 10.1017/s0033291702006074. 12214795

[R30] Kincheloe JL, Mclaren P, 2011. Rethinking critical theory and qualitative research. In: Hayes K, Steinberg SR, Tobin K (Eds.), Key Works in Critical Pedagogy,pp. 285–326. 10.1007/978-94-6091-397-6_23. SensePublishers.

[R31] Klar M, Kasser T, 2009. Some benefits of being an activist: measuring activism and its role in psychological well-being. Polit. Psychol 30 (5), 755–777. 10.1111/j.1467-9221.2009.00724.x.

[R32] Knight Steele C, 2021. When the Black lives that matter are not our own: Digital Black feminism and a dialectic of self and community. Fem. Media Stud 21 (5), 860–863. 10.1080/14680777.2021.1949370.

[R33] Lee S-H, Cole SW, Kim Y, Choi I, 2024. Personality and the Conserved Transcriptional Response to adversity: a moderating role of meaning in life. Psychoneuroendocrinology 160, 106859. 10.1016/j.psyneuen.2023.106859.

[R34] Mehl MR, Raison CL, Pace TWW, Arevalo JMG, Cole SW, 2017. Natural language indicators of differential gene regulation in the human immune system. Proc. Natl. Acad. Sci 114 (47), 12554–12559. 10.1073/pnas.1707373114. 29109260 PMC5703282

[R35] Moieni M, Irwin MR, Seeman TE, Robles TF, Lieberman MD, Breen EC, Okimoto S, Lengacher C, Arevalo JMG, Olmstead R, Cole SW, Eisenberger NI, 2020. Feeling needed: effects of a randomized generativity intervention on well-being and inflammation in older women. Brain Behav. Immun 84, 97–105. 10.1016/j.bbi.2019.11.014. 31759092 PMC7010547

[R36] Nam S, Dunton GF, Ordway MR, Ash GI, Jeon S, Vlahov D, Whittemore R, Nelson LE, Sinha R, Nunez-Smith M, Granger DA, 2020. Feasibility and acceptability of intensive, real-time biobehavioral data collection using ecological momentary assessment, salivary biomarkers, and accelerometers among middle-aged African Americans. Res. Nurs. Health 43 (5), 453–464. 10.1002/nur.22068. 32856310 PMC8985242

[R37] Ortlipp M, 2015. Keeping and using reflective journals in the qualitative research process. Qual. Rep 10.46743/2160-3715/2008.1579.

[R38] Perera S, 2023. Misogynoir transformed: black women’s digital resistance. Synoptique 10 (1), 98–101,138.

[R39] Petersen EE, 2019. Racial/ethnic disparities in pregnancy-related deaths—United States, 2007–2016. MMWR. MMWR (Morb. Mortal. Wkly. Rep.) 68. 10.15585/mmwr.mm6835a3. PMC6730892 31487273

[R40] Reed RG, Hillmann AR, Nation M, Braksator S, Sigler K, 2024. Remote dried blood spot collection for inflammatory markers in older adults is feasible, reliable, and valid. Brain Behav. Immun 120, 545–553. 10.1016/j.bbi.2024.07.001. 38971206 PMC11781373

[R41] Rodgers BL, Cowles KV, 1993. The qualitative research audit trail: a complex collection of documentation. Res. Nurs. Health 16 (3), 219–226. 10.1002/nur.4770160309. 8497674

[R42] Rogers M, Allen D, 2024. Embedding reflexivity in social work research through the critical reflexive framework. Br. J. Soc. Work 54 (4), 1753–1772. 10.1093/bjsw/bcad245.

[R43] Snodgrass JG, Bendeck S, Zhao KX, Sagstetter S, Lacy MG, Nixon C, Branstrator JR, Arevalo JMG, Cole SW, 2022. Social connection and gene regulation during the COVID-19 pandemic: divergent patterns for online and in-person interaction. Psychoneuroendocrinology 144, 105885. 10.1016/j.psyneuen.2022.105885. 35961191 PMC9335856

[R44] Steger MF, Frazier P, Oishi S, Kaler M, 2006. The meaning in life questionnaire: assessing the presence of and search for meaning in life. J. Counsel. Psychol 53 (1), 80–93. 10.1037/0022-0167.53.1.80.

[R45] Szymanski DM, Goates JD, Strauss Swanson C, 2023. LGBQ activism and positive psychological functioning: the roles of meaning, community connection, and coping. Psychol. Sex. Orientat. Gend. Divers 10 (1), 70–79. 10.1037/sgd0000499.

[R46] Uchôa E, Vidal JM, 1994. Medical anthropology: conceptual and methodological elements for an approach to health and disease. Cad. Saúde Pública 10 (4), 497–504. 10.1590/S0102-311X1994000400010. 14676936

[R47] Vines AI, Baird DD, Stevens J, Hertz-Picciotto I, Light KC, McNeilly M, 2007. Associations of abdominal fat with perceived racism and passive emotional responses to racism in African American women. Am. J. Publ. Health 97 (3), 526–530. 10.2105/AJPH.2005.080663. PMC1805011 17267721

[R48] Witeska-Młynarczyk A, 2015. Critical medical anthropology – a voice for just and equitable healthcare. Ann. Agric. Environ. Med 22 (2), 385–389. 10.5604/12321966.1152099. 26094543

[R49] Wnuk A, Oleksy T, Gambin M, Woźniak-Prus M, Łyś A, Holas P, 2023. Collective action mitigates the negative effects of COVID-19 threat and anti-abortion restrictions on mental health. Soc. Sci. Med 335, 116225. 10.1016/j.socscimed.2023.116225. 37729820

[R50] Wolk MW, Strecher VJ, Hill PL, 2024. Considering the wellbeing correlates of activist purpose. J. Happiness Stud 25 (7), 108. 10.1007/s10902-024-00815-x.

[R51] Woods-Giscombé CL, 2010. Superwoman Schema: african American women’s views on stress, strength, and health. Qual. Health Res 20 (5), 668–683. 10.1177/1049732310361892. 20154298 PMC3072704

[R52] Woods-Giscombe C, Robinson MN, Carthon D, Devane-Johnson S, Corbie-Smith G, 2016. Superwoman schema, stigma, spirituality, and culturally sensitive providers: factors influencing African American women’s use of mental health services. Journal of Best Practices in Health Professions Diversity: Research, Education and Policy 9 (1), 1124–1144. 33043323 PMC7544187

